# Prison psychiatry and faking symptoms

**DOI:** 10.1192/j.eurpsy.2021.90

**Published:** 2021-08-13

**Authors:** V. Tort Herrando

**Affiliations:** Unitat Salut Mental Brians 1, Parc Sanitari Sant Joan de Deu, Sant Esteve Sesrovires., Spain

**Keywords:** Faking symptoms, malingering, feigned symptoms and prison

## Abstract

Faking symptoms is not an unusual finding in psychiatry; As a such is not a symptoms o sign of mental disorder; we could say that lying is frequent in the normal life of people. In psychiatry, in the community has been widely reported (), mainly related to legal psychiatry (getting some social benefits, avoiding legal obligations, etc). From forensic psychiatry, this topic have a special relevance as they have more serious consequences (to avoid prison, child custody, etc)(Resnick 2003, Gunn 2014). Another topic of paramount importance is that in psychiatry we have not complementary examinations (RMN, TAC, blood tests, etc) that help to discard some symptoms. Some test are used for detecting feigned symptoms as SIMS, The most important psychological episodes in prison are those related to disruptive / bizarre behaviour, suicide ideation and psychotic symptoms that create a great nuisance to Prison Governors. To get an accurate diagnosis is very important because this could have a psychiatric approach or a prison sanction. Sometimes a previous feigned symptom does not mean to have new episode with psychiatric symptomatology that should be treated. In this paper we´ll focus in the prison psychiatry (that probably include all the situations that has been describe above.) and to give some clinical tips to deal with this kind of situation in the everyday work and casualty job.

**Disclosure:**

No significant relationships.

